# Preparation of Vanillin-Taurine Antioxidant Compound, Characterization, and Evaluation for Improving the Post-Harvest Quality of Litchi

**DOI:** 10.3390/antiox12030618

**Published:** 2023-03-02

**Authors:** Hafiz Umer Javed, Ruofan Liu, Cuijin Li, Sixia Zhong, Jiechang Lai, Murtaza Hasan, Xugang Shu, Li-Yan Zeng

**Affiliations:** School of Chemistry and Chemical Engineering, Zhongkai University of Agriculture and Engineering, Guangzhou 510225, China

**Keywords:** vanillin schiff base, antioxidant acticity, post-harvest browning, litchi

## Abstract

Litchi’s post-harvest pericarp browning is one of the main constraints that drastically affect its visual attributes and market potential. Therefore, the vanillin-taurine Schiff base (VTSB) compound prepared from natural compounds of vanillin and taurine exhibited higher DPPH-radical-scavenging invitro antioxidant activity than vanillin. VTSB first-time report to mitigate the postharvest browning of litchi fruit. In this study, litchi fruits were dipped in 0.3 mM (based on pre-experiment) VTSB solution and stored at 25 ± 1 °C for six days to examine their effects on browning and postharvest quality. Fruit treated with VTSB had lower levels of browning degree (BD), browning index (BI), weight loss, soluble quinone (SQ), relative electrolyte leakage (REL), and malondialdehyde (MDA) than control fruit. Additionally, total anthocyanins and phenolic concentrations, Total soluble solids (TSS), and 2,2-diphenyl-1-picrylhydrazyl-free radical scavenging activity (DPPH-RSA) were preserved higher in VTSB-treated litchi fruit. The levels of Ascorbate peroxidase (APX), Superoxide dismutase (SOD), and Catalase (CAT) were higher in treated fruit, whereas polyphenol oxidase (PPO) and Peroxidase (POD) were decreased during the postharvest period. This study suggested that VTSB would be very useful for different post-harvest problems in the fruit and vegetable industry.

## 1. Introduction

Litchi is an economical fruit crop with pinkish to bright red skin and edible aril having high juice content and a pleasing taste [1]. Its non-climatic nature prevents it from further ripening after harvest, thus litchi pericarp quickly turns brown within one–two days of being stored at room temperature which downgrades cosmetic quality and reduces marketability. The pericarp browning in harvested litchi fruit has also been associated with higher water loss, susceptibility chilling injury, rapid post-harvest senescence, an energy imbalance, fungal decay, and higher reactive oxygen species (ROS) generation [2,3,4]. It has been reported that oxidation of the phenolics in the litchi pericarp markedly leads to the formation of quinones, and direct polymerization into brownish pigments linked with accelerated anthocyanin degradation which consequently promotes the post-harvest browning [5]. PPO and POD enzymes are known to be key markers that have a significant function in enzymatic browning during storage due to the oxidation reaction of phenolics [5,6,7].

In order to overcome the major constraint of enzymatic browning in the litchi industry, it has been reported that the application of SO_2_ at a commercial scale significantly reduced the rapid oxidation reaction of phenolics that are responsible for litchi pericarp browning. Nevertheless, SO_2_ fumigation has some adverse side effects, including altered fruit flavor and health risks (carcinogenic effects) for packhouse employees and customers, as well as, negative consequences on quality and safety [8]. Accordingly, there is a dire need for developing food-safe technology for the quality preservation of litchi fruit during the post-harvest supply chain. Therefore, a natural, safe, eco-friendly, and antioxidant product needs to be explored that preserves the fruit’s quality while being secure for both customers and workers in the packaging industry.

In the last two decades, several natural compounds with high antioxidant capacity such as apple polyphenols [4], α-Lipoic acid [9], salicylic acid [10], melatonin [11], tea seed oil [12], oxalic acid [13], and *Aloe vera* gel coating [14] have been found effective for the alleviation of pericarp browning and maintaining the storage quality of litchi fruit by reducing ROS and preserving higher antioxidant systems [12,13]. Therefore, it is essential to develop a natural antioxidant product that has engaged researchers in order to protect agricultural products from environmental and safety hazards.

Plant phenols are well-known natural compounds with a phenolic hydroxy structure, such as vanillin (4-hydroxy-3-methoxy benzaldehyde), which showed significant anti-oxidative properties, and thus resulted in a safe food additive [15,16,17]. Vanillin was reported to keep the activity of defense-related enzymes constant, including POD, PPO, and phenylalanine ammonia-lyase (PAL) in tomato fruit [18], it protected the grapes from postharvest rot caused by yeast, mold, and *Botrytis cinerea*, and extended the post-storage quality [19,20]. On the other hand, an amino acid derived Schiff base attracted more attention due to its special biological activities including antioxidant, and antibacterial [21,22,23]. Taurine is a naturally occurring amino acid that is widely distributed in human tissues and organs and was used as a supplementation or additive in food. Moreover, taurine also has higher antioxidant activity and is utilized as a functional agent in medicine [24,25]. Notably, there is an aldehyde group inside vanillin that could form a Schiff base with taurine. Therefore, both vanillin and taurine were combined by the Schiff base reaction for producing a superior antioxidant product, which helps to preserve the fruit without any environmental or health concerns.

Expectedly, the preliminary in vitro anti-oxidant evaluation of VTSB through the DPPH-RSA test showed a positive result. Currently, there is sporadic research work on employing plant phenols as applications regarding fruit preservation, and to the best of our knowledge, vanillin-taurine Schiff base has not been tested to preserve the post-harvest fruit quality, while VTSB has antioxidant potential and environmental protection, which fulfill the requirement for food safety. In this study, the VTSB was utilized to overcome the main post-harvest browning in the litchi pericarp and to explore the enzymatic mechanism. The newly synthesized compound will be helpful for managing browning in litchi fruit and preserving its quality by substituting traditional antibrowning treatments with VTSB compounds.

## 2. Materials and Methods

### 2.1. General Information for Preparation of Schiff Compounds

All reagents including taurine and vanillin were purchased from commercial providers (Shanghai Macklin Biochemical Technology; Shanghai, China) and employed without additional purification. ^1^H NMR spectra were acquired on a Bruker spectrometer (Avance NEO 600 MHz; Bruker, Switzerland) operating at 400 MHz D_2_O, and chemical shifts were reported in ppm.

### 2.2. Preparation of VTSB (E)-3-((4-Hydroxy-3-methoxybenzylidene)amino) Propane-1-Sulfonate Compound

**Figure 1:**
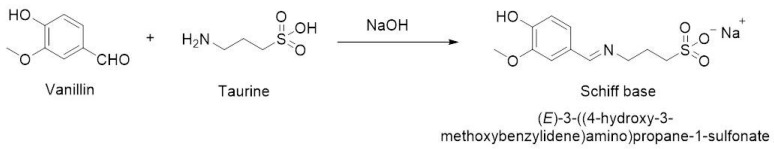
The synthesis of Vanillin-taurine Schiff-Based (*E*)-3-(4-hydroxy-3-methoxybenzylidene) amino) propane-1-sulfonate) compound.

For synthesizing the VTSB compounds, 250.5 g (2 mol) of taurine and 128 g (3.2 M) of NaOH were added to 300 mL of methanol under 40 °C till the mixture was clear. Then, 300 mL of methanol containing 304.3 g (2 M) of vanillin was added gradually into the reaction mixture and heated to reflux and the eventual yellow solid started to be produced sequentially. After 30 min of continuous refluxing and stirring, the reaction was completed. It was then allowed to cool to room temperature. The yellow solid substance was separated by filtering (Figure 1), yielding 472 g (yielded in 80%), which was structurally identified as VTSB by 1H NMR.

### 2.3. In Vitro Antioxidant Activity Evaluation

The DPPH radical scavenging test is a rapid technique for invitro screening and evaluation of antioxidants. DPPH radical-scavenging test was performed following a protocol of Hai-Yun and Shuo-sheng, 2022 with minor modifications. The antioxidant activity was measured on UV-Vis at 517 nm, and the result was calculated using the formula below:

Radical-scavenging activity (%) = [1 − (As − Ag)/Ae] × 100

Ae: Absorbance of DPPH solution in EtOH at 517 nm.

Ag: Absorbance of the sample solutions in EtOH at 517 nm.

As: Absorbance of DPPH and sample solution in EtOH at 517 nm.

### 2.4. Litchi Sampling

Litchi fruits (*Litchi chinensis* Sonn. cv. “Guiwei”) were hand-harvested at commercial maturity from a commercial orchard “Zhou Huang Bai” in Baiyun, Guangzhou, Guangdong, China. The well-known and traditional Guiwei (GW) cultivar is distinguished by its pink-red color. The litchi fruit was first sorted and graded at the orchard before being transferred to the Zhongkai University of Agriculture and Engineering within 2 h.

#### Experimental Treatments

The litchi fruits were re-sorted at the workstation and divided into two groups after being examined for uniformity in size, shape, and color, as well as free from any blemishes and disease signs. In total, 500 fruits were used in this experiment, which included two treatments (25 fruits per treatment, each with 3 replicates) and four storage periods. The dipping duration for both treatments was five min: one group was treated with 0.3 mM VTSB compounds, while the second was dipped in distilled water. The final concentration (0.3 mM) was determined based on a preliminary study that employed 0.3, 0.6, and 0.8 mM. Litchi fruit was treated and then allowed to air dry for 60 min at room temperature. After that, 25 treated fruits were packed in polypropylene bags with 6 holes and stored for 0, 2, 4, and 6 days at ambient conditions (25 ± 2 °C; RH 80–90%).

### 2.5. Browning Index (BI), Browning Degree (BD), Weight Loss

Based on Sivakumar and Korsten’s [2] scale descriptions, the browning of litchi skin was visually evaluated. The scale has five categories: 1 (no browning), 2 (1–2 brown spots), 3 (25%), 4 (50%), and 5 (75–100%). Lin et al. [26] adopted the methodology to determine the litchi pericarp’s BD. The first step was to ground a 1 g sample of litchi skin in an extraction solution made up of 4 mL of methanol (60%), phosphate buffer (0.1 M; pH 6.8), and polyvinylpyrrolidone (2%). The homogenized sample was centrifuged at 15,000× *g* for 15 min to extract the supernatant. Finally, the supernatant measured the BD at 450 nm and was expressed as OD450 g^−1^ fresh weight (FW). The weight of the litchi fruit was determined using an electronic balance after drying from treatments, prior to storage, and at the end of each storage period. The weight loss was calculated using the formula (WL % = W1 − W2/W1 × 100) and expressed as a percentage.

### 2.6. H_2_O_2_ and O_2_^•−^ Contents

The Velikova and Loreto method was slightly modified to measure H_2_O_2_ concentration [27]. The peel sample (1 g) was centrifuged at 12,000× *g* for 15 min after extraction with 0.1% TCA (3 mL). Afterward, 0.5 mL of the filtrate was diluted with 1 mL of KI (1 M) and 10 mM L^−1^ of phosphate buffer. At 390 nm, the solution absorbance was measured and reported as μmolkg-1. Yang et al. [28] methodology was followed with a few minor adjustments to establish the O_2_^•−^ production rate. Briefly, 1 g of the pericarp from a litchi fruit was well mixed with a solution containing 50 mM of phosphate buffer (3 mL; 7.8 pH) and 1 percent of polyvinylpyrrolidone. The assay was then centrifuged (10,000× *g*) for 15 min at 4 °C. Monitoring NO_2_ generation from hydroxylamine in the existence of O_2_^•−^ and comparing the results with a standard curve were used to compute the production of O_2_^•−^ content, and calculated as nmol min^−1^ kg^−1^.

### 2.7. Relative Electrolyte Leakage (REL), Malondialdehyde (MDA) Content

REL was measured according to the protocol adopted by Chen et al. [29]. Peel discs of the same size were removed from 10 litchis, mixed with 20 mL of deionized water, and then left at room temperature (25 °C) for 30 min. Afterward, the electric conductivity (EC) meter was employed to measure the initial reading (Lt). After the initial reading, the sample was heated in a water bath for 15 min. The last value (Lo) was taken after the heated solution had cooled, and the following equation was used to calculate REL as a percentage.

REL(%) = Lt/Lo × 100

With minor modifications, MDA was estimated according to Zhang et al. [30] methodology. Litchi peel was properly homogenized in trichloroacetic acid (5 mL) and centrifuged (10,000× *g*; 20 min). Then, 2 mL of supernatant was collected and used to react with 2 mL of thiobarbituric acid. First, the assay mixture was boiled (100 °C for 15 min) and then cooled and centrifuged (5000× *g*) for 15 min. For MDA calculation, the absorbance was noted at 600, 532, and 450 nm and stated as nmol kg^−1^.

MDA (nmol kg^−1^) = [6.45 (A532 − A600) − 0.56 × 450] × Vt × Vr/(Vs × m)

where m is the mass sample; Vt expresses the extracted solution volume; Vr denotes the reaction mixture’s volume; Vs is the total extracted solution volume that contains the reaction substance

### 2.8. Soluble Quinone (SQ), Total Anthocyanin Content (TAC), Total Phenolic Content (TPC)

Banerjee et al. [31] assay was used to calculate the content of SQ. One gram of litchi peel was homogenized entirely in 10 mL methanol before being centrifuged (12,000× *g*; 20 min). The supernatant was then obtained and directly utilized for absorbance at 437 nm, representing OD437 g^−1^ FW. Zheng and Tian’s [13] approach was utilized to determine the TAC. The 10 g of litchi skin was extracted with 15 mL of 0.15% HCl and 95% methanol (15: 85), and the extract’s absorbance was recorded using a UV-1800 UV-VIS spectrophotometer at 530, 620, and 650 nm (Shimadzu, Kyota, Japan). The formula used to calculate the TAC was ΔA g^−1^ = (A530 − A620) 0.1(A650 − A620).

The Folin-Ciocalteu reagent assay used as an extraction solution to determine the TPC was the same as that used previously by Ainsworth and Gillespie [32], and the absorbance was calibrated at 735 nm. For TPC calculation, gallic acid’s standard curve was plotted and the concentration was given in mg kg^−1^.

### 2.9. Total Soluble Solids (TSS), Titratable Acidity (TA), Ascorbic Acid (AA)

TSS was calculated using a digital refractometer and was represented as Brix. TA was determined through titration with NaOH (0.1 N) and reported as a percentage of malic acid [33]. The amount of ascorbic acid (AA) in the litchi juice was determined using a titration assay with 2,6-dichlorophenol-indophenol [34].

### 2.10. Enzymatic Activities

DPPH-RSA was analyzed in litchi pericarp using the procedures described by Brand-Williams et al. [35]. Firstly, extracting the litchi skin in methanol, and then 50 µL of the extract was mixed with DPPH (0.1 mmol L^−1^). Then, the solution was left to stand for 30 min at room temperature (25 °C) in a dark room, and the absorbance was gauged at 517 nm. This process was carried out three times, and the DPPH-RSA was computed as a percentage.

Using the protocols and directions of commercial kits (Nanjing Jiancheng Bioengineering Institute), the activity of enzymes such as superoxide dismutase (SOD), polyphenol oxidase (PPO), ascorbate peroxidase (APX), peroxidase (POD), and catalase (CAT) were measured in litchi peel tissues.

### 2.11. Statistical Analysis

The data were represented as the mean ± standard error (SE). The SPSS statistical program, version 16.0 (SPSS, Inc., Chicago, IL, USA), was used to analyze the data. To compare the means, an Independent-Samples T-test was utilized. The factorial design (LSD) of the study was used to examine the significance of the vanillin treatment and storage period at level *p* ≤ 0.05.

## 3. Results and Discussion

### 3.1. The Preparation of VTSB and In Vitro Antioxidant Capacity

**Figure 2:**
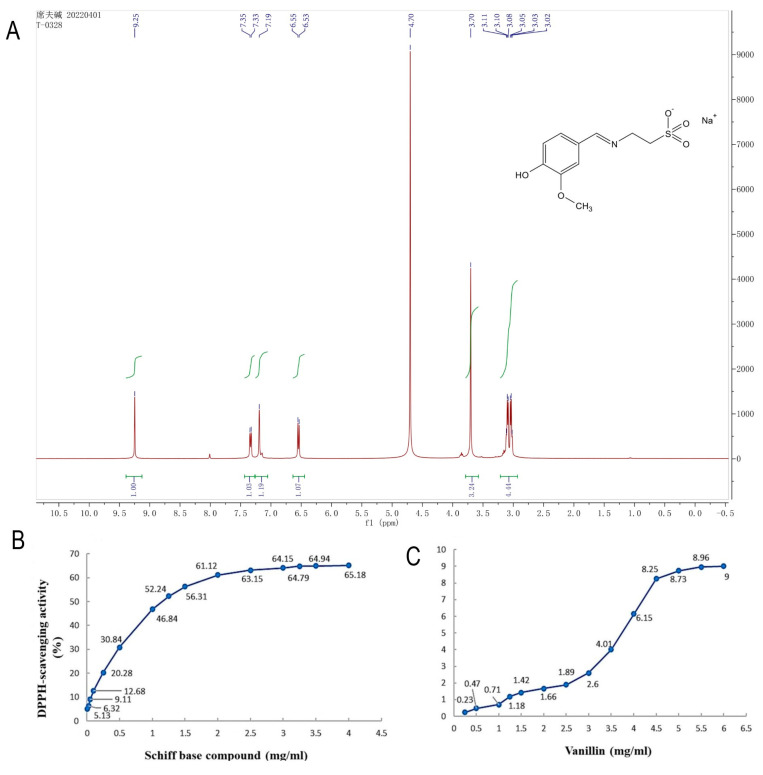
Characterization of vanillin-taurine Schiff-Based compound using HNMR (**A**); Invitro antioxidant activity of newly synthesized vanillin-taurine Schiff-Based compound (**B**); Vanillin antioxidant activity (**C**).

The research commenced with the synthesis of vanillin Schiff base. Vanillin was added to the methanol solution containing NaOH and taurine. After stirring the reaction mixture under reflux for 30 min, the expected VTSB molecule was formed successfully as shown in Figure 2A which was structurally identified by ^1^H NMR.

The antioxidant activity of vanillin and Schiff base compound (vanillin and taurine) at different concentrations was measured (Figure 2B,C). The DPPH-RSA improved as the concentration of the Schiff base solution increased, and the subsequent activity was steady until it stabilized. The greatest scavenging rate of 65.18% was recorded at a concentration of 4.0 mg/mL of Schiff base solution, whereas the rate of DPPH-RSA increased and approached 50% at concentrations lower than 1.5 mg/mL (Figure 2A). Regarding vanillin (3.0 to 5.0 mg/mL), DPPH-RSA concentration was quickly raised. Vanillin has a very low level of antioxidant activity, as evidenced by the outcome that when the concentration of the vanillin reference solution is between 5.0 and 6.0 mg/mL, DPPH-RSA slowly increased, tended to stabilize, and the rate did not exceed 10% (Figure 2B). The findings showed that compounds based on the Schiff reaction (vanillin and taurine) have greater antioxidant activity at lower concentrations as compared to simple vanillin.

### 3.2. Optimization of VTS-Compound

**Figure 3:**
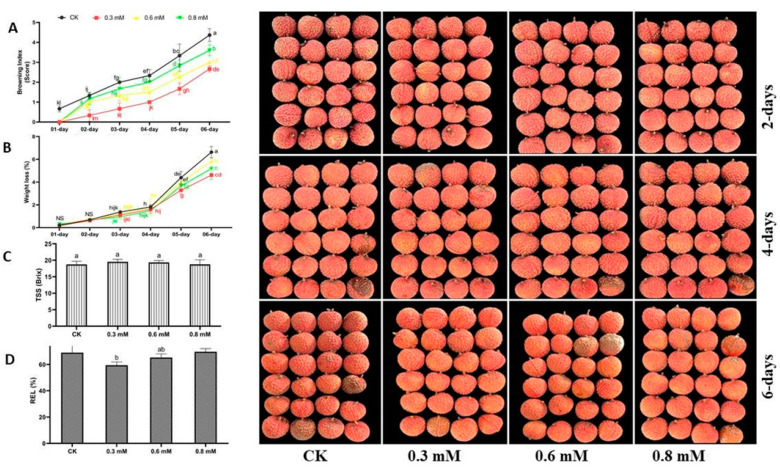
Effect of different concentrations of vanillin-taurine Schiff-Based compound on litchi fruit quality during storage. Browning degree (**A**); Weight loss (**B**); TSS (**C**); Relative electrolyte leakage (**D**); Relative electrolyte leakage; According to the LSD test, different letters (a, b, c, d, e, f, g, h, i, j, k, l and m) reflect statistical differences at the 0.05 level; *n* = 3.

The first step was to optimize the VTSB compound based on the measurement of the following variables: pericarp browning, weight loss, litchi fruit quality (TSS; TA and ascorbic acid), BD, and REL. The 0.3 mM treatment was found to be more effective as compared to 0.6 mM and 0.8 mM (Figure 3).

### 3.3. BI, BD, Weight Loss

**Figure 4:**
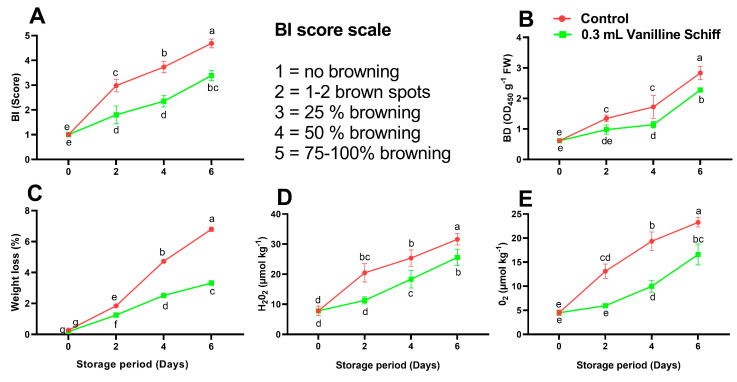
Effect of vanillin-taurine Schiff-Based compound on Browning index (**A**); Browning degree (**B**); Weight loss (**C**); H_2_O_2_ content (**D**); O_2_ content (**E**); According to the LSD test, different letters (a, b, c, d, e, f and g) reflect statistical differences at the 0.05 level; *n* = 3.

Compared to untreated (control) fruit, vanillin-treated fruit displayed a considerably lower BI throughout the storage period (Figure 4A). Only a small proportion of the litchi fruit in the vanillin-treated group had BI on day two. However, a minor increase was found on day four. Besides, BI quickly grew in the fruit that was not treated, and the fruit completely turned brown on day six (Figure 4A). BD values increased with storage time and were observed consistently significantly lower in vanillin-treated fruit than in non-treated fruit throughout the study (Figure 4B). Post-harvest browning had a negative impact on the overall appearance of fruit and led to substantial economic losses as a result of the lack of marketability [8]. Litchi fruit turns brown after harvest because anthocyanin pigments are oxidized due to desiccation.

In this study, litchi weight loss (WL) increased significantly as storage time progressed. Furthermore, the increase in WL was substantially lower in the fruit treated with vanillin application as compared to the control (Figure 4C). On day six of storage, the WL percentage in the vanillin group was markedly lower (2.04-fold) than in the control fruit. Water loss from the fruit’s surface reduces consumer acceptance as well as market value [8]. Vanillin was found to be effective in reducing water loss and preserving the quality of litchi fruit in this study.

### 3.4. H_2_O_2_ and O_2_^•−^ Contents

During storage, ROS (O_2_ and H_2_O_2_) accumulation rates eventually increased in both the control and VTSB treatments; nevertheless, the accumulated levels of these two ROS in the treated fruit were significantly lower than those in the non-treated fruit (Figure 4D,E). The production of ROS is frequently associated with loss of membrane integrity and other stress-related situations, such as browning and senescence during the storage of litchi fruit [36]. Therefore, in our study, the VTSB compound may decrease H_2_O_2_ and O_2_^•−^ formation because it minimized membrane peroxidation and senescence.

### 3.5. SQ, TAC, TPC

**Figure 5:**
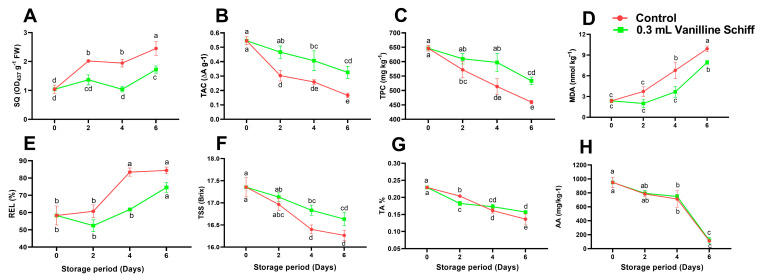
Effect of vanillin-taurine Schiff-Based compound on Soluble quinone (**A**), total anthocyanin content (**B**), total phenolic content (**C**), Malondialdehyde (**D**), Relative electrolyte leakage (**E**), Total soluble solid (**F**), Titratable acidity (**G**) and Ascorbic acid (**H**) of litchi fruit during storage; According to the LSD test, different letters (a, b, c and d) reflect statistical differences at the 0.05 level; *n* = 3.

The principal catalyst for the enzyme-induced browning of fruits and vegetables is the transformation of phenolic chemicals into quinones, which are then polymerized to form brown, red, or black pigments. In both treatments, the concentration of SQs was much higher than it was on day 0 of the experiment (Figure 5A). However, post-harvest treatment of VTSB compounds significantly reduced the rise in SQ compared to the control. A direct correlation existed between the generation of SQs and processes that led to browning, with larger production of SQs being the cause of browning [37].

Overall, TAC decreased gradually in both treatments during storage (Figure 5B). However, the TAC of the non-treated fruit decreased at a faster rate than that of the VTSB-treated fruit during storage. Anthocyanin is responsible for the red coloration of the litchi pericarp [38]. On the other hand, it degrades quickly after harvest. The distinctive red color of the litchi fruit is thought to be essential for visual appearance and economic potential [2]. The breakdown of the vacuole leads to the degradation of anthocyanin, which is spurred on by the breakdown of cellular compartmentation. Anthocyanins are eventually degraded by enzymes [39]. In this study, VTSB compounds play an essential role in slowing the degradation process of anthocyanin and ultimately attracting the consumer.

Regardless of the treatments, a significant decline in TPC occurred in the litchi pericarp tissues as storage days progressed (Figure 5C). This decrease was, however, more pronounced in the control treatment than in the fruit treated with VTSB. TPC levels in litchi fruit treated with VTSB were 16.14% higher than those in the control. Reduced TPC probably occurs from oxidation, which ultimately causes the browning of the litchi fruit [7]. As a result, fruit treated with VTSB had higher TPC values, which might play a significant role in slowing down the oxidation process in litchi fruit.

### 3.6. REL and MDA Content

The VTSB compounds significantly lowered the production of MDA content compared to the control (Figure 5D). From day two to six, there was a noticeable difference in MDA level between VTSB compounds and control fruit. The results showed that the control had a 10.36% higher MDA concentration on day six than the VTSB treatment. The storage times and treatments also significantly affected REL. Overall, REL increased throughout the study for both the control and the VTSB-treated fruits (Figure 5E). Leakage is a key indicator of membrane integrity, and loss of membrane integrity can lead to the decompartmentation of enzymes and substrates, which can contribute to the pericarp browning of litchi [40]. However, fruit treated with VTSB significantly improved membrane integrity and displayed inferior REL than non-treated fruit. On day four of storage, the VTSB treatment significantly reduced REL by 35.06% compared to the control group. (Figure 5E). According to Sun et al. [41], oxidative stress is the cause of cell membrane damage, which raises the MDA and REL levels in litchi pericarp [4,42]. By maintaining antioxidant activity and delaying post-harvest senescence, treated fruit can protect its cellular membranes from lipid peroxidation and membrane disruption. Therefore, low REL and MDA content in VTSB application was typically influenced by preserved membrane integrity, greater antioxidant activities, and less oxidative damage and senescence.

### 3.7. TSS, TA and AA

TSS continuously dropped during the six days of storage. However, compared to untreated control fruit, the TSS remained higher as in VTSB-treated fruit during storage (Figure 5F). The taste is closely associated with the changes in the TSS of litchi fruit during the post-harvest period [39]. Post-harvest senescence causes TSS to decline, which eventually downgrades the eating quality of fruit [6]. The application of VTSB compounds has been found to prevent pericarp discoloration and reduce water loss as a result of delayed senescence. Accordingly, the application of VTSB significantly prevented senescence and preserved TSS during storage.

TA gradually decreased as storage time increased (Figure 5G). TA content normally decreased in litchi fruit with longer storage times [7]. Fruit senescence occurs after harvest, the TA level is reduced due to oxidation [43]. On day two, VTSB-treated fruit showed a lower TA content than untreated control fruit (Figure 5G). However, the difference in TA between the two treatments on days two and four was non-significant. Regarding ascorbic acid levels in fruit during the entire period of storage, there was a non-significant difference between the VTSB treatment and control group (Figure 5H). Nevertheless, the rate of reduction in AA level considerably accelerated with storage time. According to Mditshwa et al. [44], the oxidative degradation of AA causes its content to decrease normally during storage.

### 3.8. Antioxidant Activity

**Figure 6:**
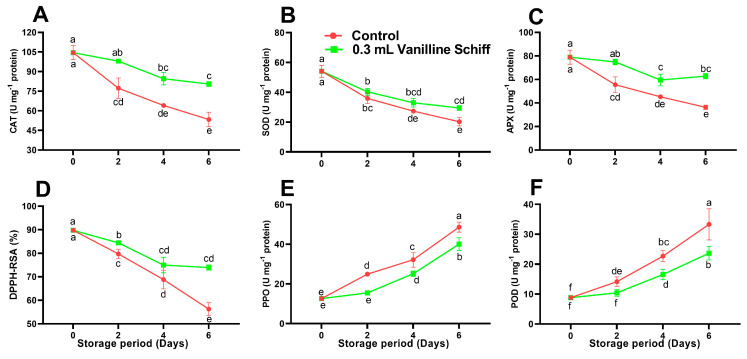
Effect of vanillin-taurine Schiff-Based compound on enzymatic and non-enzymatic antioxidant activity of litchi fruit during storage. CAT (**A**); SOD (**B**); APX (**C**); DPPH-RSA (**D**); PPO (**E**); POD (**F**); According to the LSD test, different letters (a, b, c, d, e and f) reflect statistical differences at the 0.05 level; *n* = 3.

DPPH-RSA is generally used to calculate non-enzymatic antioxidant activity in plant tissues, and its level typically decreases as a result of increased free radical production in litchi peel tissues after extensive post-harvest storage [7]. The outcomes indicated that the VTSB compound has good potential to keep higher DPPH-RSA due to a lower generation of free radicals. Therefore, VTSB-treated litchi fruit displayed increased DPPH-RSA, likely as a consequence of a lower rate of senescence and H_2_O_2_ and O_2_^•−^ content during the post-harvest period. Furthermore, VTSB treatment demonstrated higher content than the control during storage (Figure 6D); a particularly large differential of 31.14% was detected on day six. It is thought that litchi peel with a higher DPPH-RSA will help to reduce oxidative stress [34].

SOD, CAT, and APX have been linked to plant stress responses associated with senescence as well as direct and indirect ROS scavenging [45]. SOD, CAT, and APX activity in both vanillin-treated and untreated control litchi fruit showed a decreasing trend with progress in the storage period. The APX and CAT activities remained constant on days four and six of the storage. Fruit treated with the VTSB compound had significantly increased antioxidant enzyme activity (SOD, CAT, and APX) throughout storage than the control fruit (Figure 6A–C). Antioxidative enzymes like SOD, CAT, and APX detoxify various free radicals and minimize oxidative damage, which helps to prevent the litchi fruit from browning. Therefore, upregulation of the aforementioned enzymes is essential to decrease the prevalence of browning in litchi fruit [34].

According to Tomas-Barberan and Espin [46], the POD and PPO enzyme are reported to be associated with the oxidation of phenolic compounds. In the litchi fruit, phenolics and anthocyanidin produced by the enzyme anthocyanase may act as substrates to be oxidized by POD and PPO to o-quinone and other substances, resulting in pericarp browning [40]. As far as the activities of POD and PPO are concerned, the overall storage study showed an increasing trend irrespective of treatments (vanillin-treated litchi and untreated control litchi fruit) (Figure 6E,F). Furthermore, the fruit that had been dipped in the novel vanillin solution showed lower activity as compared to the water-dipped fruit. Vanillin-dipped litchi fruit showed 40.73% and 21.42% lower POD and PPO activities, respectively, than the control after six days of storage. Prooxidant enzymes (POD and PPO) are present in organelles (besides the phenolics) that oxidize phenolics and cause the browning of litchi fruit [14]. Therefore, to prevent the harvested litchi fruit from turning brown, the activity of the above enzymes should be reduced [4]. The dipping treatment preserves membrane integrity and inhibits POD and PPO activities and creates a barrier among the phenolics and enzymes that helps to reduce phenolics oxidation and prevents membrane rupture [47]. Novel vanillin compounds might have displayed reduced PPO and POD enzyme activity as a result of conserved compartmentation.

## 4. Conclusions

The newly synthesized compound VSTB showed remarkable in vitro antioxidant activity, with a DPPH-scavenging rate of 65.18%. The 0.3 mM VTSB application enhances the three-day marketable shelf life of litchi fruit which might be due to the suppression of browning and senescence. The VTSB treatment can prevent litchi pericarp browning due to its potential to suppress anthocyanin, phenolics, and flavonoid degradation as well as minimize membrane leakage (REL, H_2_O_2_, and O_2_) as compared to the control. Furthermore, it maintains antioxidant properties, both enzymatic and non-enzymatic, which may improve in scavenging ROS. As a result, the application of VTSB might be a feasible and secure approach to preventing pericarp browning and improving post-harvest storage of litchi fruit. The effectiveness of VTSB compounds as a post-harvest preservative on various fruits, including litchi fruit texture and quality will be examined in further investigations.

## Data Availability

Data is contained within the manuscript.
